# Impact of Epstein-Barr virus co-infection on natural acquired *Plasmodium vivax* antibody response

**DOI:** 10.1371/journal.pntd.0010305

**Published:** 2022-08-03

**Authors:** Michelle H. F. Dias, Luiz F. F. Guimarães, Matheus G. Barcelos, Eduardo U. M. Moreira, Maria F. A. do Nascimento, Taís N. de Souza, Camilla V. Pires, Talita A. F. Monteiro, Jaap M. Middeldorp, Irene S. Soares, Cor J. F. Fontes, Francis B. Ntumngia, John H. Adams, Flora S. Kano, Luzia H. Carvalho

**Affiliations:** 1 Instituto René Rachou/FIOCRUZ Minas, Belo Horizonte, Minas Gerais, Brazil; 2 Center for Global Health and Infectious Diseases Research, Department of Global Health, College of Public Health, University of South Florida, Tampa, Florida, United States of America; 3 Instituto Evandro Chagas, Secretaria de Vigilância em Saúde, Ministério da Saúde (IEC/SVS/MS), Belém, Pará, Brazil; 4 Department of Pathology, Free University Medical Center, Amsterdam, The Netherlands; 5 Faculdade de Ciências Farmacêuticas, Universidade de São Paulo (USP), São Paulo, Brazil; 6 Julio Müller School Hospital, Faculdade de Medicina, Universidade Federal de Mato Grosso, Cuiabá, Mato Grosso, Brazil; London School of Hygiene and Tropical Medicine Faculty of Infectious and Tropical Diseases, UNITED KINGDOM

## Abstract

**Background:**

The simultaneous infection of *Plasmodium falciparum* and Epstein-Barr virus (EBV) could promote the development of the aggressive endemic Burkitt’s Lymphoma (eBL) in children living in *P*. *falciparum* holoendemic areas. While it is well-established that eBL is not related to other human malaria parasites, the impact of EBV infection on the generation of human malaria immunity remains largely unexplored. Considering that this highly prevalent herpesvirus establishes a lifelong persistent infection on B-cells with possible influence on malaria immunity, we hypothesized that EBV co-infection could have impact on the naturally acquired antibody responses to *P*. *vivax*, the most widespread human malaria parasite.

**Methodology/Principal findings:**

The study design involved three cross-sectional surveys at six-month intervals (baseline, 6 and 12 months) among long-term *P*. *vivax* exposed individuals living in the Amazon rainforest. The approach focused on a group of malaria-exposed individuals whose EBV-DNA (amplification of *balf-5* gene) was persistently detected in the peripheral blood (PersV_DNA_, n = 27), and an age-matched malaria-exposed group whose EBV-DNA could never be detected during the follow-up (NegV_DNA_, n = 29). During the follow-up period, the serological detection of EBV antibodies to lytic/ latent viral antigens showed that IgG antibodies to viral capsid antigen (VCA-p18) were significantly different between groups (PersV_DNA_ > NegV_DNA_). A panel of blood-stage *P*. *vivax* antigens covering a wide range of immunogenicity confirmed that in general PersV_DNA_ group showed low levels of antibodies as compared with NegV_DNA_. Interestingly, more significant differences were observed to a novel DBPII immunogen, named DEKnull-2, which has been associated with long-term neutralizing antibody response. Differences between groups were less pronounced with blood-stage antigens (such as MSP1-19) whose levels can fluctuate according to malaria transmission.

**Conclusions/Significance:**

In a proof-of-concept study we provide evidence that a persistent detection of EBV-DNA in peripheral blood of adults in a *P*. *vivax* semi-immune population may impact the long-term immune response to major malaria vaccine candidates.

## Author summary

In the Amazon rainforest, both *Plasmodium vivax* and Epstein-Barr virus (EBV) infections are common, yet the Burkitt’s lymphoma (BL) is rare, despite an association between endemic BL with chronic *P*. *falciparum* exposure. Nevertheless, the influence of EBV infection on malaria immunity remains undetermined. Here, we investigated for the first time whether continuous detection of EBV-DNA in the peripheral blood of adults exposed to *P*. *vivax* could impact the antibody response to blood-stage malaria vaccine candidates. The methodological approach involved 12-month follow-up among *P*. *vivax*-exposed Amazonian classified as persistent EBV-DNA carriers (PersV_DNA_) and an age-matched group with no viral DNA detection (NegV_DNA_); groups were further differentiated based on profile of viral antibodies (mainly IgG VCA-p18). Collectively, our findings demonstrated that antibody levels against *P*. *vivax* antigens were in general lower in the PersV_DNA_ group as compared with NegV_DNA_. More significant differences were observed to a novel vaccine candidate (DEKnull-2) whose antibody response were previously associated with broadly neutralizing *P*. *vivax* antibodies. Differences between groups were less pronounced with *P*. *vivax* antigens associated with seasonal changes in the antibody responses. In this conceptual study, we provide evidence that long-term detection of EBV in peripheral blood may impact immune response to major malaria vaccine candidates.

## Introduction

The impact of malaria infection is greater on populations living in the poorest regions of the globe, where sanitary conditions are precarious, leaving the population subject to possible co-infections with other infectious agents, including parasites [1,2], bacteria [3,4] and viruses [5,6]. Consequently, in malaria endemic areas, the simultaneous infection with multiple pathogens has implications for understanding the development of protective immunity as well as the efficacy of antimalarial vaccines [7,8]. Common co-infections include herpes viruses as most vertebrates are infected with one or more types that remain for the rest of their lives [9].

The severe consequence of co-infection involving holoendemic *Plasmodium falciparum* and the Epstein-Barr virus (EBV)—a gammaherpes virus that infects B cells and maintains latency throughout the individuals’ lifetime [10,11]—is well established, as the interaction could promote the development of endemic Burkitt’s Lymphoma (eBL) [12,13]. In Sub-Saharan Africa, eBL is the most lethal of childhood cancers, with the highest prevalence in children aged 5–9 years old who are chronically exposed to *P*. *falciparum* malaria (revised by [14,15]). Malaria appears to play multiple roles in eBL etiology, including the expansion of latently infected B-cells and the likelihood of c-myc translocation that is a hallmark of BL tumors [16–19]. Of relevance, the aggressive eBL childhood cancer seems to be exclusively linked to *P*. *falciparum* exposure, but not to other human malaria parasites [20].

Despite the compelling evidence indicating a role for *P*. *falciparum* in impaired immune responses that control EBV infection [21–23], the impact of acute EBV infection on the generation of anti-malarial immunity is uncertain [24]. Notwithstanding, rodent models of EBV demonstrate that it is possible for a primary gammaherpes virus infection to negatively modulate the generation of antimalarial immunity [7]; an outcome that was correlated with a defect on the generation of humoral immunity to a secondary malaria infection. Evidence of the immune suppressive nature of an acute EBV infection on the development of malaria immunity has also been suggested in experimentally marmosets co-infected with EBV and the quartan malaria *P*. *brasilianum* [25].

Considering the B-cell compartment as the primary niche for EBV persistence [11] and that humoral malaria immunity may be altered during EBV co-infection [7], we hypothesized here that EBV co-infection could impact on the naturally acquired antibody responses to *P*. *vivax*, the most geographically widespread human malaria parasite [26]. Taking into account the rapid spread of *P*. *vivax* drug-resistant strains [27], and the potential for relapse, progress towards the development of a *P*. *vivax* vaccine is critical (reviewed in [28]). In this perspective, studying *P*. *vivax* immunity in the context of EBV co-infections can enhance our understanding of malaria-protective immunity and progress towards the design of next-generation malaria vaccines. Here, we took advantage of a longitudinal follow-up study previously carried out in the Amazon rainforest, where *P*. *vivax* IgG responders were identified to major leading *P*. *vivax* blood-stage vaccine candidates [29–31], including the Apical Membrane Antigen-1 (AMA-1) [32]; the 19-kDa C-terminal region of the Merozoite Surface Protein-1 (MSP1-19) [33]; and the Duffy Binding Protein region II (DBPII), a key ligand involved in the main *P. vivax* reticulocyte invasion pathway [34]. Since DBPII induces both strain-specific [35], and strain-transcending antibody responses [36], we also included in this study a novel engineered DBPII construct (DEKnull-2) associated with broad DBPII antibody responses [37]. To investigate whether a persistent EBV infection could interfere with this profile of *P*. *vivax* antibody responses, we examined in the study population the presence of circulating viral DNA over the time as well as EBV antibody response to lytic viral capsid antigen -VCA, replication activator protein–ZEBRA, early diffuse antigen- EAd, and latent EBV nuclear antigen 1—EBNA-1 (revised by [38]).

## Methods

### Ethics statement

The ethical and methodological aspects of this study were approved by the Ethical Committee of Research on Human Beings from the René Rachou Institute (Reports No. 007/2006, No. 07/2009, No.12/2010, No. 26/2013 and CAAE 50522115.7.0000.5091), according to the Resolutions of the Brazilian Council on Health (CNS-196/96 and CNS-466/2012). Formal written consent was obtained from all participants, which was also obtained from the next of kin, caregivers, or guardians on the behalf of child participants.

### Area and study population

The study was carried-out in the agricultural settlement of Rio Pardo (1°46’S—1°54’S, 60°22’W—60°10’W), Presidente Figueiredo municipality, Northeast of Amazonas State in the Brazilian Amazon region. The methodological approach involved a retrospective study, in which local malaria transmission pattern was described in detail elsewhere [29,39]. In this area, malaria transmission is considered hypo to mesoendemic, and most residents were natives of the Amazon region. Inhabitants of the settlement live on subsistence farming and fishing along the small streams. In the study area, *P*. *falciparum* malaria incidence has decreased drastically in recent years, and *P*. *vivax* is now responsible for all clinical malaria cases reported [30].

### Study design and cross-sectional surveys

A population-based open cohort study was initiated in November of 2008 and included three cross-sectional surveys carried at six-months interval (baseline, 6 and 12-months), and distributed in periods of high and low malaria transmission (**S1 Fig**), as previously reported [29]. Briefly, (i) interviews were conducted through a structured questionnaire to obtain demographical, epidemiological, and clinical data; (ii) physical examination, including body temperature and spleen/liver size were recorded according to standard clinical protocols; (iii) venous blood was collected for individuals aged five years or older (EDTA, 5 mL); and (iv) examination of Giemsa-stained thick blood smears for the presence of malaria parasites by conventional light microscopy, with *P*. *vivax* infection confirmed later by a species-specific real-time PCR as described [40]. The geographical location of each dwelling was recorded using a hand-held 12-channel global positioning system (GPS) (Garmin 12XL, Olathe, KS, USA) with a positional accuracy of within 15 m. For the current study, the non-eligible criteria were (i) refusal to sign the informed consent; (ii) young children (<5 years), as children have not yet developed a robust malaria-specific antibody response [41]; (iii) pregnant women; (iv) any other morbidity that could be traced; and (v) individuals who were unable to be recruited during all three consecutive cross-sectional surveys. Initially, 360 participants were eligible to the current study. The methodological strategy further involved screening all 360 malaria-exposed individuals according to the detection of circulating EBV-DNA during the baseline and consecutive cross-sectional surveys (6-months, 12-months). For the current study, we focused on two sub-groups of malaria-exposed individuals (i) individuals whose EBV-DNA could be persistently detected in peripheral blood (PersV_DNA_) and (ii) an age-matched subgroup whose viral DNA was undetected throughout the follow-up period (NegV_DNA_) (**Table 1**).

**Table 1:** Demographic, epidemiological, and parasitological data of malaria-exposed individuals whose EBV-DNA could be detected (PersVDNA) or not (NegVDNA) in the peripheral blood during the 12-month follow-up period.

	PersV_DNA_	NegV_DNA_	*p value*
	(n = 27)	(n = 29)	
**Characteristic**			
Age, median, years *(IQR)* ^*a*^	38 (22–54)	33 (27–42)	*p = 0*.*1227*
Gender ratio, male:female	1.5/1	0.9/1	*p = 0*.*4357*
Years of malaria exposure, median *(IQR)* ^*b*^	32 (16–51)	33 (25–40)	*p = 0*.*7233*
Years of residence in Rio Pardo, median *(IQR)* ^*c*^	8 (4–10)	9 (3–14)	*p = 0*.*2326*
Riverine population, *n (%*) ^d^	4 (15%)	6 (21%)	*p = 0*.*7308*
Self-reported malaria episodes, median *(IQR)*	5 (2–11)	11 (3–21)	*p = 0*.*1410*
Acute *Plasmodium vivax* infection, *n (%)* ^*e*^			
Baseline	6 (22%)	6 (21%)	
6 months	1 (4%)	3 (10%)	
12 months	4 (15%)	5 (17%)	

As adults can have antibodies against EBV for the rest of their lives [38], plasma samples of a group of children (n = 34; median age 9 years, IQR 8–11) were used to confirm the sensitivity and specificity of the EBV-peptides used in serological assays.

### Recombinant blood stage *P*. *vivax* proteins and IgG antibodies detection

#### DBPII-related antigens

***DBPII-Sal1***, a recombinant Duffy binding protein region II (DBPII) including amino acids 243–573 of the Sal-1 reference strain [42], and recombinant ***DEKnull-2***, an engineered DBPII immunogen [37], were expressed as a 39kDa 6xHis fusion protein, properly refolded, as previously described [37,43]. ***MSP1-19 antigen*.** The 19-kDa C-terminal region of the Merozoite Surface Protein-1 of *P*. *vivax* (MSP1-19), which represents amino acids 1616–1704 of the full-length MSP-1 polypeptide, has been described elsewhere [44]. ***AMA-1 antigen*.** The ectodomain of *P*. *vivax* Apical Membrane Antigen-1 (AMA-1, encompassing amino acids 43 to 487, were produced as previously described [45]. To enable purification, MSP1-19 and AMA-1 constructs were also produced as carboxyl-terminal 6xHis-tag fusion proteins from *Escherichia coli* and *Pichia pastoris*, respectively. ***Conventional Enzyme-Linked Immunoassays (ELISA) for P*. *vivax IgG antibodies*** was carried out using *P*. *vivax* blood-stage recombinant proteins as previously described [30], with serum samples at a dilution of 1:100. Recombinant proteins were used at a final concentration of either 3 μg/mL (DBPII and DEKnull-2) or 1 μg/mL (MSP1-19 and AMA-1). For each protein, the results were expressed as ELISA reactivity index (RI), calculated as the ratio of the mean optical density (OD at 492 nm) of each sample to the mean OD plus three standard deviations of negative control plasma samples from 30 individuals living in a nonendemic area of malaria (Belo Horizonte, Minas Gerais, Brazil) and who have never been exposed to malaria transmission (unexposed volunteers). Values of RI > 1.0 were considered seropositive.

### EBV antigens and serostatus

EBV-specific antibodies were detected using 4 synthetic peptides covering immunodominant epitopes of the viral capsid antigen P18 (VCA-p18 [BFRF3]), EBV nuclear antigen 1 (EBNA1 [BKRF1]), early diffuse antigen complex (EAd-p45/52 [BMRF1]) and BZLF1-encoded replication activator protein of EBV (Zebra [BZLF1]) [46,47]. All synthetic peptides were kindly provided by Dr. J. M. Middeldorp (VU University Medical Center, Amsterdam, Netherland). For the assessment of the levels of antibodies to lytic (VCA-p18, EAd and Zebra) and latent EBV antigens (EBNA1), we used synthetic peptide-based ELISA assays as described [48,49]. Briefly, each peptide was used at final concentration of 1 μg/mL with plasma samples diluted 1:100. IgM (VCA, Zebra and EAd) and IgG (VCA and EBNA-1) reactivities were determined using commercial anti-human IgM and IgG secondary antibodies conjugated to horseradish to peroxidase (HRP) (Sigma-Aldrich). For each experiment, plasma samples from subjects with or without a history of mononucleosis infection who had previously been screened for the presence or absence of EBV-specific antibodies were included as positive (EBV seropositive, n = 8) or negative controls (seronegative, n = 5), respectively. As recommended by the original protocol [46], all OD 450 values were normalized by subtracting the value for 1:100-diluted EBV-negative sera used in duplicate in each ELISA run. Receiver-operating characteristic (ROC) curves was used to determine optimal cutoff points for each peptide (**S2 Fig**). Based on the area under the ROC curve (AUC) the follow ELISA’s cutoff were established: (i) 0.38 for VCA IgG (82% sensitivity; 83% specificity), (ii) 0.20 for EBNA-1 IgG (75% sensitivity; 100% specificity), (iii) 0.43 for VCA IgM (82% sensitivity; 100% specificity), (iv) 0.37 for ZEBRA IgM (90% sensitivity; 100% specificity) and (v) 0.44 for EA-d IgM (85% sensitivity; 100% specificity).

### EBV DNA detection by real-time PCR

The PCR primers for this assay were previously selected in the single-copy BALF-5 gene encoding the viral DNA polymerase [50]; the upstream and downstream primer sequences were 5′-CGGAAGCCCTCTGGACTTC-3′ and 5′-CCCTGTTTATCCGATGGAATG-3′, respectively, with a fluorogenic probe (5′-TGTACACGCACGAGAAATGCGCC-3′) with a sequence located between the PCR primers. Detectable DNA from EBV was identified by a real-time PCR assay as previously described [51,52]. Briefly, DNA from whole blood samples collected in EDTA was extracted using Purogene blood core kit B (Qiagen, Minneapolis, MN, USA). The PCR reaction was performed using a mixture containing 1μL of DNA, 0.2 μM each primer, 0.1 μM fluorogenic probe, and 5 μL of TaqMan Master Mix (PE Applied Biosystems), and the PCR cycle was performed as follows: 2 min at 50°C, 10 min at 95°C, and 40 cycles of 15 s at 95°C and 1 min at 60°C. The TaqMan Master mix (PE Applied Biosystems) was used for all reactions. For all PCR analyses, water was used as negative control, and B958 and P3HR1 viral DNA were used as positive controls. The B958 and P3HR1 viral strains were kindly provided by Dr. Talita A. F. Monteiro (Federal University of Pará, PA, Brazil) and were described elsewhere [53]. Samples were defined as negative if the CT values exceeded 40 cycles.

### Quantification of EBV-DNA copies by the digital droplet PCR (ddPCR)

The ddPCR assays were prepared using the same primers and probes used in qPCR, with a total of 22 μL per reaction containing ddPCR reagents (10 μL of the Bio-Rad 1X ddPCR Super Mix [no dUTP], 900 nM of forward and reverse primers, and 250 nM of the probe), and 2 μL of the DNA template. Initially ddPCR was carried out in a Bio-Rad Droplet Generator with a Droplet Digital PCR System used to automatically generate droplets. To optimize the PCR annealing temperature, a temperature gradient of 54–63°C was used. An annealing temperature of 55°C provided better separation between the positive and negative droplets for EBV. Endpoint PCR assays were performed using the following cycling parameters: enzyme activation at 95°C for 10 min, followed by 40 cycles of denaturation at 94°C for 30s, and primer annealing at 55°C for 1 min. The results were analyzed using a Bio-Rad Droplet Reader. A threshold to promote better droplet separation of low amplitude fluorescence was determined at 3,000 RFU for EBV assays based on the droplet separation limit of blank assays. Target quantification was expressed as copies/μL of the ddPCR reaction.

### Statistical analysis

A database was created using Epidata software (http://www.epidata.dk). The graphics and the statistical analysis were performed using GraphPad Prism version 9.1.2—GraphPad Software, La Jolla California USA. Receiver operating characteristic curves (ROC) analysis was used to determine optimal Cut-off values for EBV peptides in ELISA assays. For the statistical analyses, the antibody response was defined either as a binary categorical variable (the proportion of seropositive individuals) or as a continuous variable (the levels of antibody response); differences in two medians were tested by Mann-Whitney test and Kruskal-Wallis when there are two or more medians; differences in proportions were evaluated by chi-square test or Fisher’s exact test, as appropriated; and correlations between *P*. *vivax* and EBV antibody responses were examined by Pearson’s or Spearman’s matrices. In all analysis, a significance level of 5% was considered, i.e., values of *P* < 0.05.

## Results

### Characteristic of *P*. *vivax* malaria-exposed groups

At the time of the first cross-sectional survey, 123 (34%) out of 360 Amazonian individuals initially eligible for the study had detectable EBV-DNA in the peripheral blood. Further surveys (6- and 12-month later) identified 27 out of 123 individuals as persistent viral DNA carriers, i.e., individuals whose EBV-DNA was amplified (*balf-5* gene) from the peripheral blood during all follow-up period (PersV_DNA_). In parallel, we selected a group of 29 age-matched malaria-exposed individuals with no detectable viral DNA in the peripheral blood (NegV_DNA_) (**Table 1**). Demographic, parasitological, and epidemiological variables were comparable between groups. Accordingly, most individuals were adults with similar proportion of male: female, and their age basically corresponding to their lifetime exposure to malaria in the Amazon area (medians of 32 and 33 years for PersV_DNA_ and NegV_DNA_, respectively). In this long-term malaria exposed individuals, few acute malaria infections were detected during the follow-up period (all *P*. *vivax*, as detected by microscopy and/or species-specific PCR assay). (**Table 1**).

### EBV serostatus of *P*. *vivax* malaria-exposed groups

**Fig 1:**
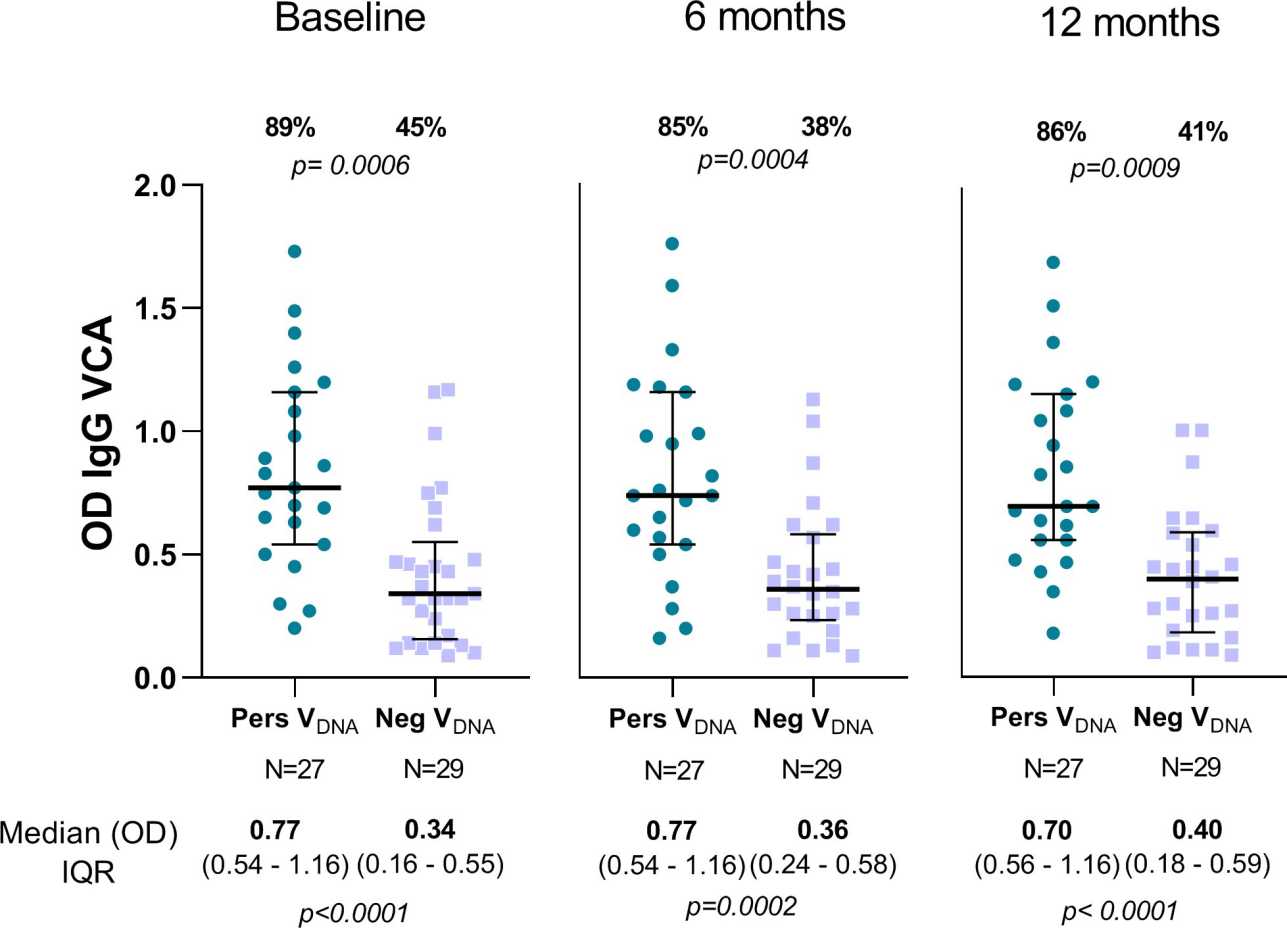
Profile of IgG antibody response against EBV capsid antigen p18 (VCA-p18) in individuals whose EBV-DNA could be detected (PersV_DNA_) or not (NegV_DNA_) over the follow-up period. For each graphic, individual datapoints were expressed as ELISA absorbance (OD) and shown here as a scatter dot plots with lines showing the median with interquartile range (IQR). Numbers represented in the top and bottom of graphics represent the proportion of responders (%) and median (OD) with IQR values, respectively; p-values for significant difference between groups were included and calculated as described in methods. The proportion of responders was determined by considering an OD > 0.38 as ELISA-positive response (S2 Fig). All raw data are available in the S1 Table.

In these immunocompetent adults, all individuals were seropositive for at least one EBV peptide during the follow-up period (**S3 Fig)**. Despite of that, individuals categorized as PersV_DNA_ had a much broader EBV antibody response than NegV_DNA_ group; specifically, while 42% of EBV-DNA carriers respond to all five EBV-ELISA markers, only 15%of NegV_DNA_ group responded to these markers (chi-square test = 4.276; *p = 0*.*038*). A similar profile of response was detected over the time (**S3 Fig**) From the panel of 4 EBV antigens, only IgG response to the lytic antigen VCA-p18 showed a clear differentiation between PersV_DNA_ and NegV_DNA_ groups (**Fig 1**). At enrollment, while 89% of PersV_DNA_ had a positive IgG antibody response to VCA-p18, only 45% of NegV_DNA_ had IgG VCA-p18 (chi-square test; *p = 0*.*0006*). Of interest, the difference in response between groups remained constant throughout the follow-up period, i.e., 85% vs. 38%, p = 0.0004; and 86% vs. 41%, p = 0.0009, for PersV_DNA_ and NegV_DNA,_ respectively). The levels of antibodies to VCA-p18 were also significantly different between groups (Mann-Whitney test), with levels ranging from 0.70 to 0.77 for PersV_DNA_ and from 0.34 to 0.40 for NegV_DNA_ groups **(Fig 1).** Considering the individual EBV antibody response over time, we observed low individual variability, i.e., high/low VCA-p18 responders retained their antibody profile throughout the follow-up period (S4 Fig).

**Fig 2:**
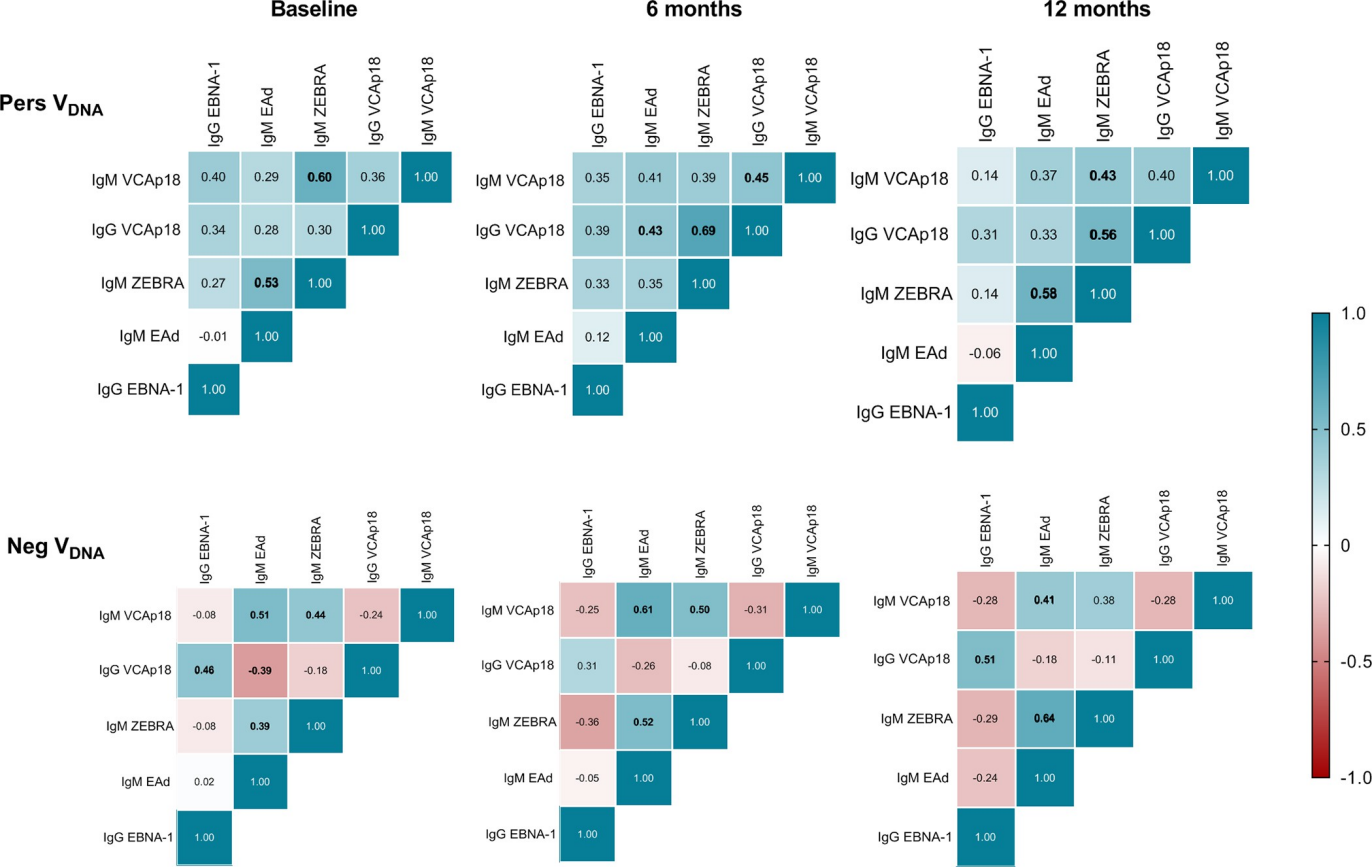
Pairwise correlations between EBV antibody levels in individuals whose EBV-DNA could be detected (PersV_DNA_) or not (NegV_DNA_) over the follow-up period. Clustering was based on the Spearman correlation coefficient for assays measuring anti-EBV antibodies in plasma samples [IgM (VCA-p18, Zebra and EAd-p45/52) and IgG (VCA-p18 and EBNA-1)]. Matrix heatmaps were shown for each cross-sectional survey (baseline, 6- and 12-months), with top and bottom panels representing PersV_DNA_ and NegV_DNA_ groups, respectively. Positive correlations shown in blue and negative correlations shown in red, with numbers in bold statistically significant differences.

Although IgM antibodies to EBV lytic antigens (VCA-p18, EAd-p45/52 and Zebra) did not show any differences between the study groups, at the individual level, a positive IgM response for one EBV antigen was related to positivity for the others (**S5 Fig**); of note, this IgM response profile was sustained throughout the study period. Further, we compared the pattern of humoral antibody responses to all EBV antigens between PersV_DNA_ and NegV_DNA_ (**Fig 2**). In the PersV_DNA_ group, there was a tendency of positive correlation between EBV-specific antibodies, specially to IgM antibodies. On the contrary, NegV_DNA_ group was characterized by a predominance of negative antibody correlations to several EBV antigens.

### *Plasmodium vivax* antigen-specific antibody responses in persistent viral DNA carriers

**Fig 3:**
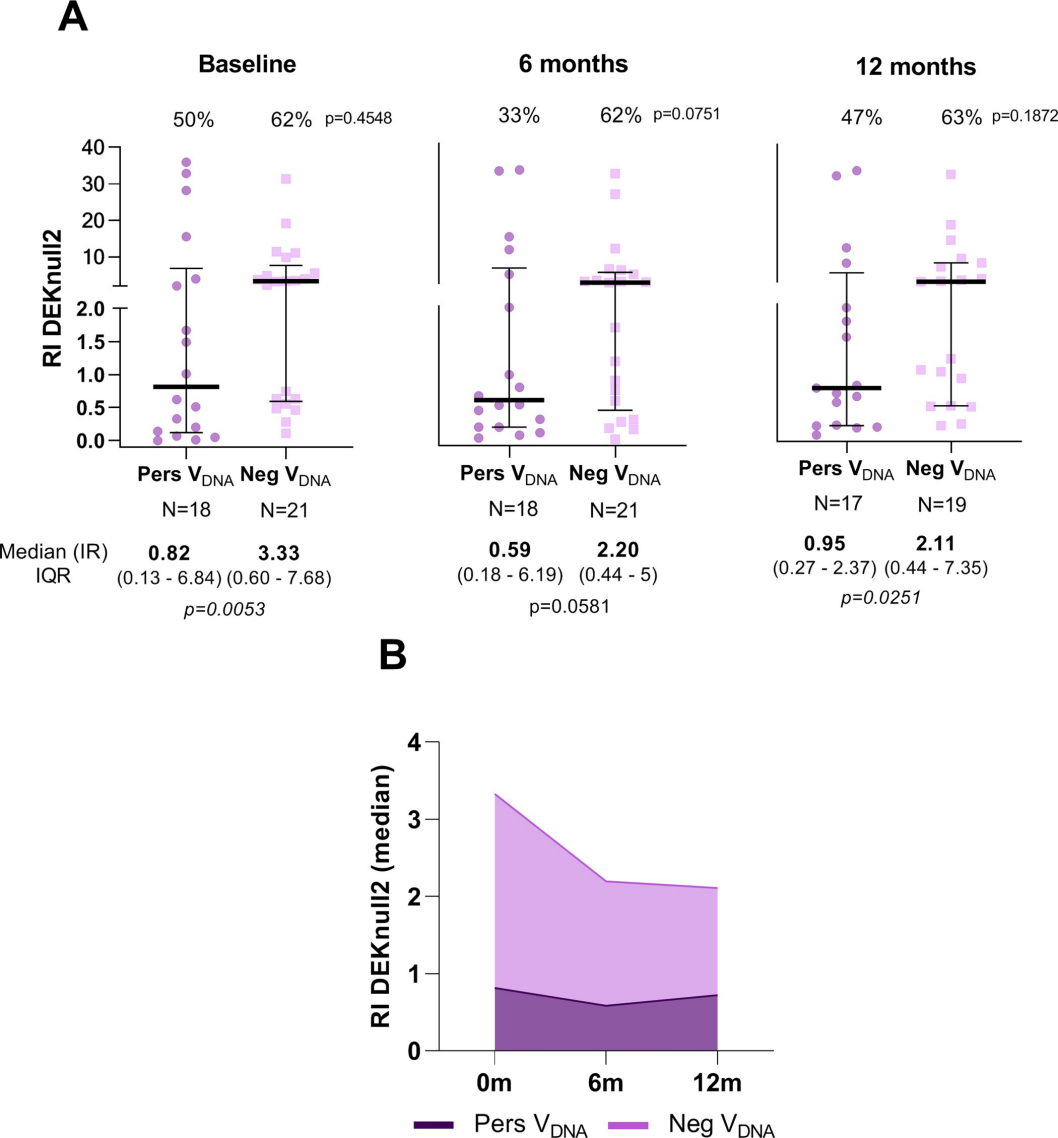
Profile of IgG antibody response against the surface-engineered DEKnull-2 vaccine of *P*. *vivax* in individuals with (PersV_DNA_) or without (NegV_DNA_) persistent viral DNA over the follow-up period. In **A,** results are shown by cross-sectional surveys (baseline, 6- and 12-month), with individual data points expressed as ELISA Reactivity Index (RI) and shown here as a scatter dot plots with lines showing the median with interquartile range (IQR). In **B**, medians of RIs overtime for each group. Numbers in the top and bottom of each graphic represent the proportion of responders (%) and median (RI) with IQR values, respectively; p-values for significant difference between groups were included and calculated as described in methods. Reactivity Index (RI) was calculated as described in methods and RI > 1 corresponded to an ELISA-positive response.

**Fig 4:**
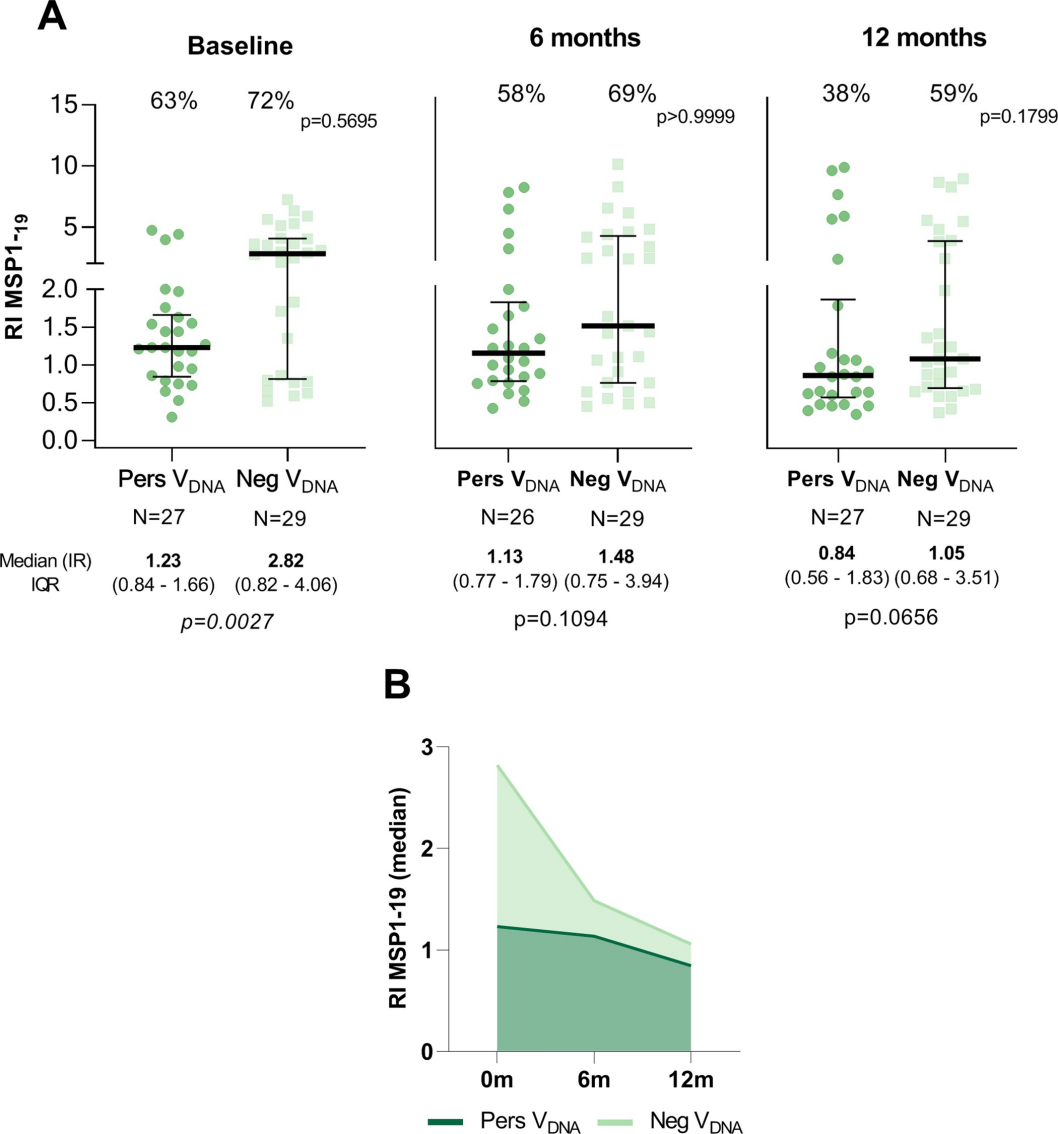
Profile of IgG antibody response against the 19-kDa C-terminal region of the Merozoite Surface Protein-1 (MSP1-19) in individuals with (PersV_DNA_) or without (NegV_DNA_) persistent viral DNA over the follow-up period. In **A,** results are shown by cross-sectional surveys (baseline, 6- and 12-month), with individual data points expressed as ELISA Reactivity Index (RI) and shown here as a scatter dot plots with lines showing the median with interquartile range (IQR). In **B,** medians of RIs overtime for each group. Numbers in the top and bottom of each graphic represent the proportion of responders (%) and median (RI) with IQR values, respectively; p-values for significant difference between groups were included and calculated as described in methods. Reactivity Index (RI) was calculated as described in methods and RI > 1 corresponded to an ELISA-positive response.

To investigate whether the continuous detection of EBV-DNA in the peripheral blood would impact the humoral response to *P*. *vivax* malaria, we evaluated antibodies to leading *P*. *vivax* blood-stage vaccine candidates (DBPII-related antigens, AMA-1 and MSP1-19). In general, there was no clear difference in the proportion of responders between groups, however, the levels of antibody response to different *P*. *vivax* antigens were significantly different between PersV_DNA_ and NegV_DNA_. The antibody response to the engineered DBPII immunogen (DEKnull-2) showed a trend towards lower antibody levels in PersV_DNA_ compared with NegV_DNA_ (**Fig 3**). Specifically, while DEKnull-2 serological reactivity index (RI) ranged from 0.59 to 0.95 for PersV_DNA_, RI values ranged from 2.11 to 3 for NegV_DNA_ group. The difference intra-group remained relatively stable overtime (Fig 3B). A similar pattern was observed with the original DBPII protein (**S6 Fig**). For *P*. *vivax* MSP1-19, the differences between the groups were less pronounced (**Fig 4**), with statistically significant differences observed only during high transmission period at the study baseline (**Fig 4**). Circulating antibodies against AMA-1 showed a similar pattern of response as MSP1-19 (**S7 Fig**).

In general, antibodies against *P. vivax* blood antigens did not correlate with EBV antibody response (**S8 Fig).** A similar pattern was found for Persv_DNA_ and NegV_DNA_ overtime, except for an unexpected positive association between DEKnull2 antibodies and IgM Ead in the PersV_DNA_ **(S8 Fig).**

Finally, to investigate whether EBV-DNA copy number in the peripheral blood would influence *P*.*vivax*-specific antibodies response, a digital droplet PCR (ddPCR) was carried in consecutive samples of the PersV_DNA_ group. In general, the EBV DNA copies/μL in the PersVDNA was relatively low, range from 0.79 (baseline) to 0.84 copies/ μl at the end of the follow-up period (**S9A Fig**) and remained < 1 copy/μl in most of malaria-exposed individuals (**S9B Fig**). While the EBV-DNA copy number correlated with VCA-p18 IgG antibodies (**S9C Fig**), the EBV-DNA copies “per se” did not influenced in *P*.*vivax*-specific antibodies (**S10 Fig**).

## Discussion

In the Amazon rainforest both malaria and EBV infections are common, yet Burkitt’s lymphoma (BL) is rare [54]; this is not unexpected as endemic BL (eBL) is linked to *P*. *falciparum* exposure, but not to other malaria parasites [20]. While *P*. *vivax* malaria is a public health problem in the Amazon, immunological outcomes of prevalent viral co-infections are often neglected [55–57]. Here, we sought to investigate whether a continuous detection of EBV-DNA in peripheral blood of *P*. *vivax* malaria-exposed adults (PersV_DNA_) could impact antibody response to key *P*. *vivax* blood-stage vaccine candidates. In this context, it is possible to speculate that a positive PersV_DNA_ status may reflect a reactive EBV persistence with associated deregulated (activated) B-cell function. Consecutive cross-sectional surveys demonstrated that levels of *P*. *vivax*-specific antibodies were in general lower in the PersV_DNA_ group compared with age-matched negative DNA carriers living in the same malaria-endemic village (NegV_DNA_). Interestingly, significant differences in antibody levels were observed against a novel DBPII immunogen, DEKnull-2, that has been associated with stronger, broader, and long-term neutralizing antibody response in the study area [31,37]. For DEKnull-2, the difference in the magnitude of the antibody response between PersVDNA and NegV_DNA_ groups ranged from two to four-fold, and these differences were maintained throughout the follow-up period. While DEKnull-2 was developed to overcome the inherent DBPII bias towards developing strain-specific immunity associated with poor immunogenicity [35], our methodological approach also included a common DBPII variant (Sal1) circulating in the Amazon area [29]. A similar profile of response was observed for the native Sal1-DBPII variant, in which less reactivity was observed in the PersV_DNA_ group. Taken together, the results suggested that long episodes of EBV-DNA detection may influence the levels of both strain-specific and strain-transcending DBPII immune responses.

With respect to more immunogenic blood stage *P*. *vivax* antigens, such as MSP1-19 [58], the difference in the magnitude of antibody response between the groups was less pronounced and observed only in samples collected at baseline (PersV_DNA_ < NegV_DNA_), when malaria transmission was more evident in the study area. Although the reasons for the difference in the profile of antibody response between *P. vivax* antigens are not known, we can speculate that local seasonal variation in malaria transmission may have played a role, as antibodies against MSP1-19 /AMA-1 may fluctuate considerably according to malaria transmission [59–61]. In accordance, we previously demonstrated that levels of MSP1-19/AMA-1 (but not DBPII-specific antigens) dropped when malaria transmission was reduced [30]. Considering that our study design involved temporal variation in malaria transmission profile (S1 Fig), it is reasonable to consider that lowest levels of these antibodies detected at 6 and 12 months of the follow-up (i.e., in the NegV_DNA_ group) may have masked the difference between PersVDNA and NegVDNA groups. Even though our sample size precluded a more robust statistical analysis, the median for MSP1-19 dropped from 3.7 (at enrollment) to 0.8–1.16 (6 and 12 months, respectively), and for AMA-1 from 2.82 to 1.48–1.05. Future studies should consider the wide range of immunogenicity between *P. vivax* blood stage proteins.

It is also worth mentioning that in this immunocompetent malaria-exposed adult population, we avoid to staging EBV infection based on serology. As we are dealing primarily with an adult population, individuals from both groups presented antibodies against at least one EBV antigens. Here, EBV serological profile was used as complementary tool to describe the serostatus of PersV_DNA_ versus NegV_DNA_ group. For that, EBV serology was built upon an extensive panel of EBV antigens, which included antigens from both latent and lytic stages [38]. More specifically, we used EBV-specific polypeptides targeting the most frequent serological markers of EBV such as the viral capsid antigens (VCA-p18), the early antigens (EAD-p47/54), Epstein–Barr nuclear antigens (EBNAs) as well as the major transcription factor of EBV expressed upon EBV lytic cycle activation (Zebra) [62]. This is a common panel used in studies involving co-infection of malaria falciparum and EBV [49,51,63]. Based on this panel of EBV antigens, it was possible to demonstrated that PersV_DNA_ had a much broader EBV antibody response than the NegVDNA group, with a significant proportion of viral DNA carriers recognizing all 5 serological markers used here. Considering individual EBV serological markers, only IgG VCA-p18 distinguished PersV_DNA_ and NegV_DNA_ groups during the follow-up study, (85–89% vs. 38–45%, respectively). Actually, measuring VCA-IgG antibodies seems to be a best single test to indicate a previous EBV infection [64]. Notwithstanding, in the PersV_DNA_ group, IgG-VCA antibodies tended to be associated with IgM antibodies to lytic antigens (VCA-p18, EAd-p45/52 and Zebra). According, only the levels of IgG VCA-p18 correlated with the number of EBV-DNA copies, suggesting that these EBV-DNA carriers may have a frequent/constant reactivation of the EBV over the months.

Intriguingly, EBV-IgM responses were relatively stable during the cross-sectional surveys (in both groups for all EBV antigens). Although the reasons for these findings are unclear, concerns have been raised about possible cross-reactivities of EBV-IgM antibodies with other antigenically related viral infections [65–67], such as CMV that is prevalent in the Amazon area [68]. Although IgM cross-reactivity could not be ruled out, the tendency of correlation with EBV-DNA may reflect constant EBV activation-triggering of B-cells; in fact, EBV persistently infecting naive or anti-EBV B-cells leading to increased IgM levels [69,70]. This is conceivable since it has been demonstrated that a large proportion of circulating B cells from healthy EBV-exposed subjects were committed to the production of IgM antibodies that were polyreactive and bound a variety of self- and exogenous antigens [71]. These findings may explain the unexpected association between DEKnull2 antibodies and IgM EAd-p45/52. Perhaps a plausible, and not mutually exclusive explanation, for our results of long-lasting IgM response is the unappreciated role of the IgM antibodies in natural infections. To diverse immunogens and pathogens, it has been demonstrated that long-lived antigen-induced IgM plasma cells demonstrate somatic mutations, contribute to long-term protection, and may persist for a lifetime [72]. Together, these findings emphasize the need to understand the role of the IgM specific antibodies in natural infections and vaccines development [73].

While the persistence of the IgM response in our adult cohort merits further investigations, it is important to clarify about the high sensitivity and specificity of the EBV-peptides used in our ELISA assays. According, in a group of 34 Amazonian children (median age 9 years, IQR 8–11) whose plasma samples were screened for anti-EBV antibodies using the same peptide-based ELISA protocol, we found that VCA-IgM and Zebra-IgM antibodies decreased over time while VCA-IgG and EBNA-IgG increased within same period of time (12-month follow-up) and remained positive throughout the observation period (**S11 Fig**). These results confirmed the typical pattern of seroconversion of young children with low socioeconomic status [38], correlating with an early EBV seroconversion for Latin America population [74].

As expected, few malaria cases were detected in our malaria semi-immune study population, which precluded our ability to evaluate an association between acute *P*. *vivax* infection and persistent detection of EBV-DNA. Although the current study was not designed to investigate whether EBV can change the course of an acute *P*. *vivax* malaria infection, scant data from Indonesia suggest that whereas EBV-DNA levels were significantly elevated in high parasitemic *P. vivax* individuals, EBV-DNA levels were not related to age, gender, or malaria symptoms [75]. To properly address the influence of the persistence of EBV-DNA detection in the course of vivax malaria, a non-immune symptomatic *P. vivax* population should be investigated that was out of the scope of the current study.

Our study has limitations that should be considered when interpreting the results. First, a relatively small number of malaria-exposed residents were eligible to participate in the study, which may have underpowered some statistical analyses. Specifically, the 12-month cohort study demonstrated that only 27 out of 360 individuals presented persistent detection of EBV-DNA in peripheral blood. At this time, it is difficult to compare the frequency of EBV-DNA detected in our study to the frequency detected in other studies. Particularly, because in immunocompetent individuals the frequency of detection of EBV-DNA in blood can vary widely, and this can be influenced by several factors, including differences in the study population, seasonality and other co-infections present in the study area [76,77]. Also, differences in DNA target, PCR protocols and biological specimens may lead to underestimate or overestimate the prevalence of DNA in blood [78]. Here, we have decided to extract DNA from the whole blood because EBV-DNA in plasma/serum may suggesting viremia; despite of that, our protocol did not allow to differentiate EBV DNA from cell free (plasma/serum) from cell-associated form (non-infectious).

Recently, an elegant study involving the blood virome of healthy individuals showed that 21% of studied blood samples had EBV expression [79]. Here, even among the small sample used, we were able to detect EBV-DNA in 34% of our samples (123 out of 360) with roughly 8% classified as long-term EBV-DNA carriers (27 out of 360). Still, large longitudinal studies are warranted to accurately determine prevalence in health individuals over time.

Additionally, the small number of participants precluded the use of a multivariate model to control confounding variables. However, our control group was adjusted by major confound variables such as age, time of exposure and time living in the study area; consequently, we believe that this did not significantly influence our findings. Notwithstanding these limitations, we are confident about the robustness of the study design, which allowed us to demonstrate for the first time that antibody levels to different *P. vivax* antigens were significantly lower in subjects with persistent EBV “DNAmia”. As the main host cell of the EBV is the human B cell (reviewed by [80]), ongoing experiments are in progress to evaluate on the role of a persistent detection of EBV-DNA in the long-term *P*. *vivax*-specific B cell response. In this proof-of-concept study, we provide evidence that a persistent detection of EBV in peripheral blood of an adult *P*. *vivax* semi-immune population may impact the long-term malaria immune response to major malaria vaccine candidates.

## Supporting information

S1 Table Individual data from the study population grouped according to the detection (PersVDNA) or not (NegVDNA) of viral DNA over the follow-up period. (XLSX)

S1 Fig Monthly-time series of malaria cases in the agricultural settlement of Rio Pardo (Amazonas, Brazil) during the study period, 2008–2009. The current study included three cross-sectional surveys at six-month intervals (Baseline, Bs; 6- and 12-months latter). Malaria cases were based on results of conventional microscopy provided by the National Malaria Surveillance System Registry (SIVEP-Malaria), with cases of *P*. *falciparum* (light blue) and *P*. *vivax* (dark blue) plotted per month.
(TIF)

S2 Fig Two-graph receiver operating characteristic curves (TG-ROC) to EBV proteins. For ELISA-detected antibodies [IgM (VCA-p18, Zebra and EAd-p45/52) and IgG (VCA-p18 and EBNA-1)], the best cutoff values was determined through sensibility and specificity calculated by TG-ROC curves in GraphPad Prism 9.2, as described in material and methods. Assay variability was measured by the coefficient of variation (CV) calculated as the standard deviation of absorbance divided by the mean; CV for positive/negative controls, respectively, were 14.9%/18.97% for IgM VCA-p18; 18.33%/ 12.18% for IgG VCA-p18; 14.34%/ 17%for IgG EBNA-1; 19.94%/ 20.21% for IgM Zebra; 11.4/ 26.9% for EAd-p45/52.
(TIF)

S3 Fig Frequency of anti-EBV antibodies according to the number of serological markers recognized by individuals whose EBV-DNA could be detected (PersV_DNA_) or not (NegV_DNA_) over the follow-up period. For each group, results were presented as the proportion of responders for one (1), two (2), three (3), four (4) or five (5) EBV serological markers [IgM (VCA-p18, Zebra and EAd-p45/52) and IgG (VCA-p18 and EBNA-1)]. All raw data are available in the S1 Table.
(TIF)

S4 Fig Profile of individual VCA-p18 antibody response in individuals with (PersV_DNA_) or without (NegV_DNA_) persistent viral DNA over the follow-up period. For both graphics, individual data points were expressed as ELISA absorbance (OD) and shown here as a scatter dot plots. Points were connected representing the individual profile over the follow-up period. The dotted line represents the cut-off value that was determined considering an OD > 0.38 as a positive ELISA response (S2 Fig) that classified subjects as low/high responders for VCA-p18.
(TIF)

S5 Fig Individual antibody response to Epstein-Barr virus (EBV) peptides (VCA-p18, ZEBRA, EAd-p45/52 and EBNA-1) during the cross-sectional surveys. Heatmaps illustrated individual antibody response of each malaria-exposed individuals classified according to the detection (PersV_DNA_) or not (NegV_DNA_) of EBV-DNA over the follow-up period. According to EBV antibody response, individuals were categorized as non-responder (negative) or responders (stratified as low, medium or high, according to EBV antibody reactivity). The missing values are recorded as blank spaces.
(TIF)

S6 Fig Profile of IgG antibody response against *P*. *vivax* Duffy Binding Protein region II (DBPII) in individuals with (PersV_DNA_) or without (NegV_DNA_) persistent viral DNA over the follow-up period. In **A,** results are shown by cross-sectional surveys (baseline, 6- and 12-month), with individual datapoints expressed as ELISA Reactivity Index (RI) and shown here as a scatter dot plots with lines showing the median with interquartile range (IQR). In **B,** medians of RIs overtime for each group. Numbers in the top and bottom of each graphic represent the proportion of responders (%) and median (RI) with IQR values, respectively; p-values for significant difference between groups were included and calculated as described in methods. Reactivity Index (RI) was calculated as described in methods and RI > 1 corresponded to an ELISA-positive response.
(TIF)

S7 Fig Profile of IgG antibody response against *P. vivax* Apical Membrane Antigen-1 (AMA-1), in individuals with (PersV_DNA_) or without (NegV_DNA_) persistent viral DNA over the follow-up period. In **A,** results are shown by cross-sectional surveys (baseline, 6- and 12-month), with individual datapoints expressed as ELISA Reactivity Index (RI) and shown here as a scatter dot plots with lines showing the median with interquartile range (IQR)). In **B,** medians of RIs overtime for each group. Numbers in the top and bottom of each graphic represent the proportion of responders (%) and median (RI) with IQR values, respectively; p-values for significant difference between groups were included and calculated as described in methods. Reactivity Index (RI) was calculated as described in methods and RI > 1 corresponded to an ELISA-positive response.
(TIF)

S8 Fig Pairwise correlations between EBV and *P*. *vivax* antibody levels in individuals with (PersV_DNA_) or without (NegV_DNA_) persistent viral DNA during the cross-sectional surveys. Clustering was based on the Spearman correlation coefficient for assays measuring anti-EBV antibodies in serum. Matrix heatmaps were shown for each cross-sectional survey (baseline, 6- and 12-months), with top and bottom panels representing Pers V_DNA_ an Neg V_DNA_ groups, respectively. Positive correlations shown in blue and negative correlations shown in orange, with numbers in bold statistically significant differences.
(TIF)

S9 Fig EBV-DNA quantification (by ddPCR) in individuals with persistent detection of the EBV DNA (PersVDNA). In **A**, the amplitude of viral DNA is shown by cross-sectional surveys (baseline, 6 and 12 months), with individual data points represented as copy number /μL. Data are shown here as scatterplots with lines showing the median with interquartile range (IQR) (values ​​shown below each cross-section). In **B**, the dots were connected representing individual variability in EBV-DNA copies overtime, and in **C**, the association between anti-VCA-p18 IgG antibodies and EBV-DNA copies, as analyzed by the Spearman’s correlation coefficient (r = 0.32 and p<0.05).
(TIF)

S10 Fig Absence of correlation between the levels of *P*. *vivax* specific antibody and EBV-DNA copy number (“DNAmia”). The correlation between antibody levels (reactivity index—RI) to *P*. *vivax* blood stage proteins—DEKnull2 (A), DBPII (B), MSP1-19 (C), AMA-1 (D)—and the viral DNA detected (copies/μL) was based on the Spearman correlation coefficient (p>0.05 for all comparison).
(TIF)

S11 Fig IgM and IgG antibody response against Epstein-Barr virus peptides in Amazonian children over time. For each EBV-peptide (VCA-p18; Zebra, EAd-p45/52 and EBNA-1) antibody response was represented by frequency of responders (bar) and magnitude of response (optical density-OD median, lines). The results represented three cross-sectional surveys carried-out at 6-month intervals, i.e., at enrollment (0m) and six (6m) and 12-month latter (12m). ELISA assays were carried out as described in the methods.
(TIF)
