# Supplementary figures and images for: Impact of Epstein-Barr virus co-infection on natural acquired Plasmodium vivax antibody response
Source: PLoS Negl Trop Dis. 2022 Aug 3;16(8):e0010305. doi: 10.1371/journal.pntd.0010305 (PMC9377613; doi:10.1371/journal.pntd.0010305)

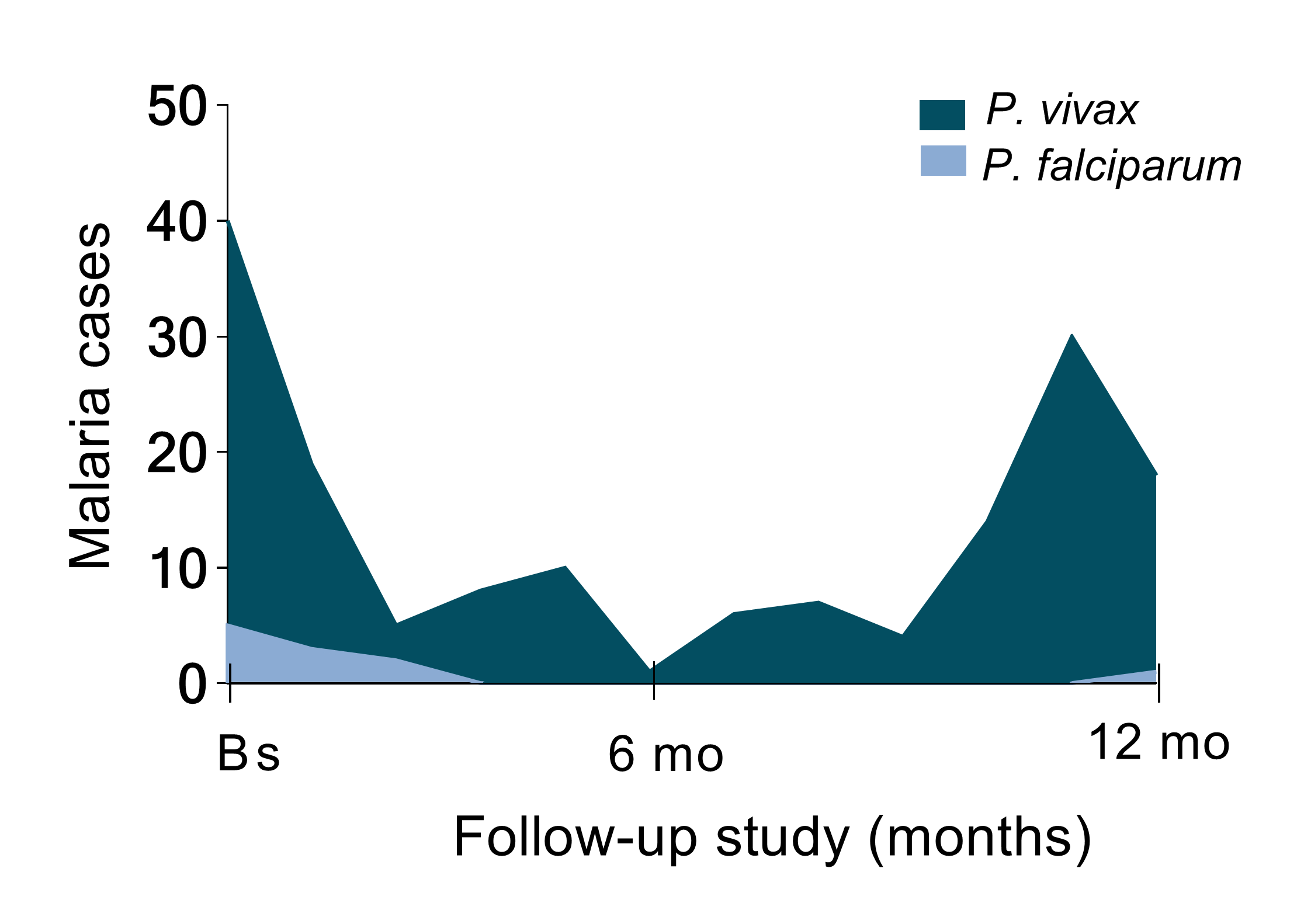

Supplement: S1 Fig — The current study included three cross-sectional surveys at six-month intervals (Baseline, Bs; 6- and 12-months latter). Malaria cases were based on results of conventional microscopy provided by the National Malaria Surveillance System Registry (SIVEP-Malaria), with cases of P. falciparum (light blue) and P. vivax (dark blue) plotted per month. (TIF) [file pntd.0010305.s002.tif]

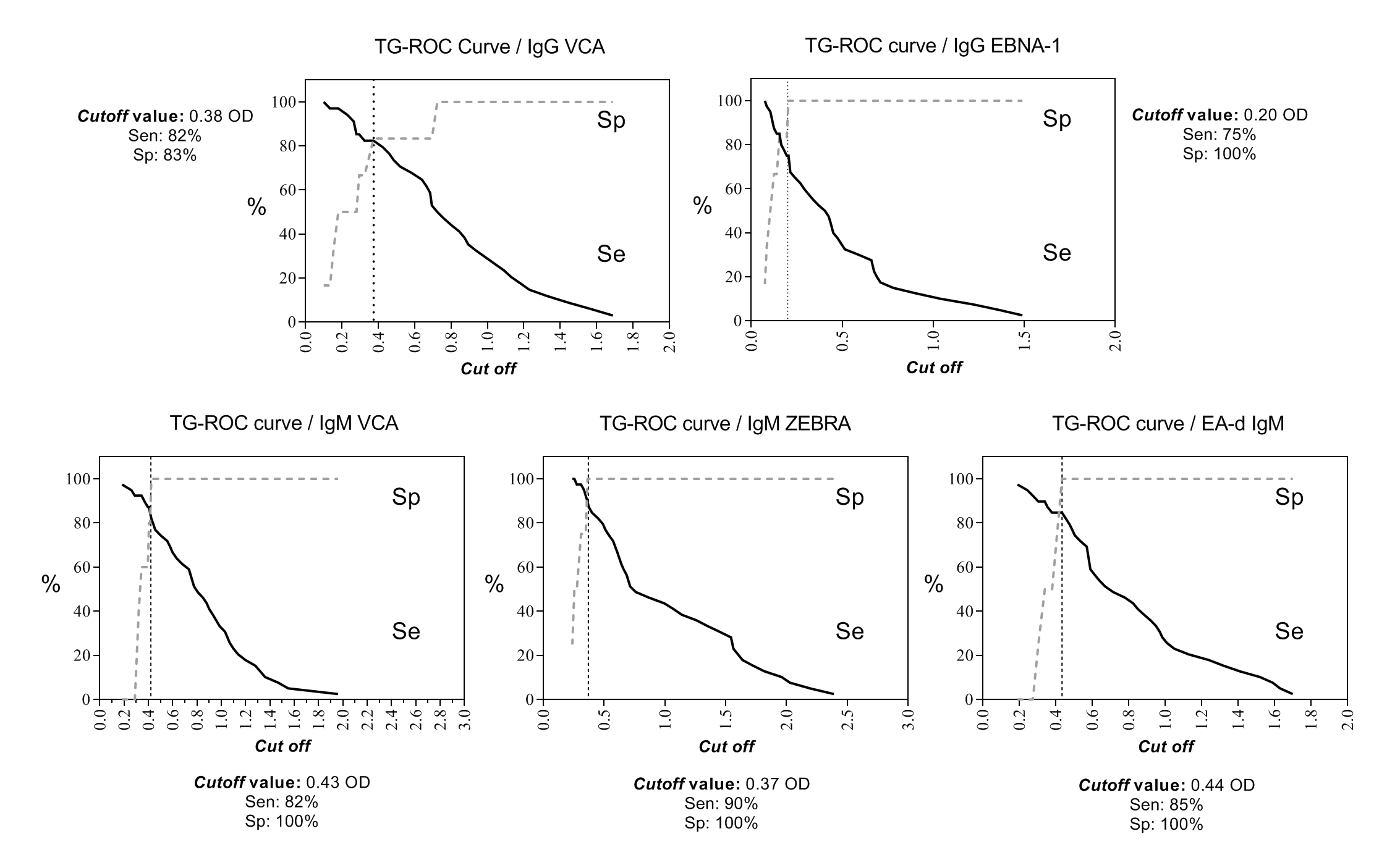

Supplement: S2 Fig — For ELISA-detected antibodies [IgM (VCA-p18, Zebra and EAd-p45/52) and IgG (VCA-p18 and EBNA-1)], the best cutoff values was determined through sensibility and specificity calculated by TG-ROC curves in GraphPad Prism 9.2, as described in material and methods. Assay variability was measured by the coefficient of variation (CV) calculated as the standard deviation of absorbance divided by the mean; CV for positive/negative controls, respectively, were 14.9%/18.97% for IgM VCA-p18; 18.33%/ 12.18% for IgG VCA-p18; 14.34%/ 17%for IgG EBNA-1; 19.94%/ 20.21% for IgM Zebra; 11.4/ 26.9% for EAd-p45/52. (TIF) [file pntd.0010305.s003.tif]

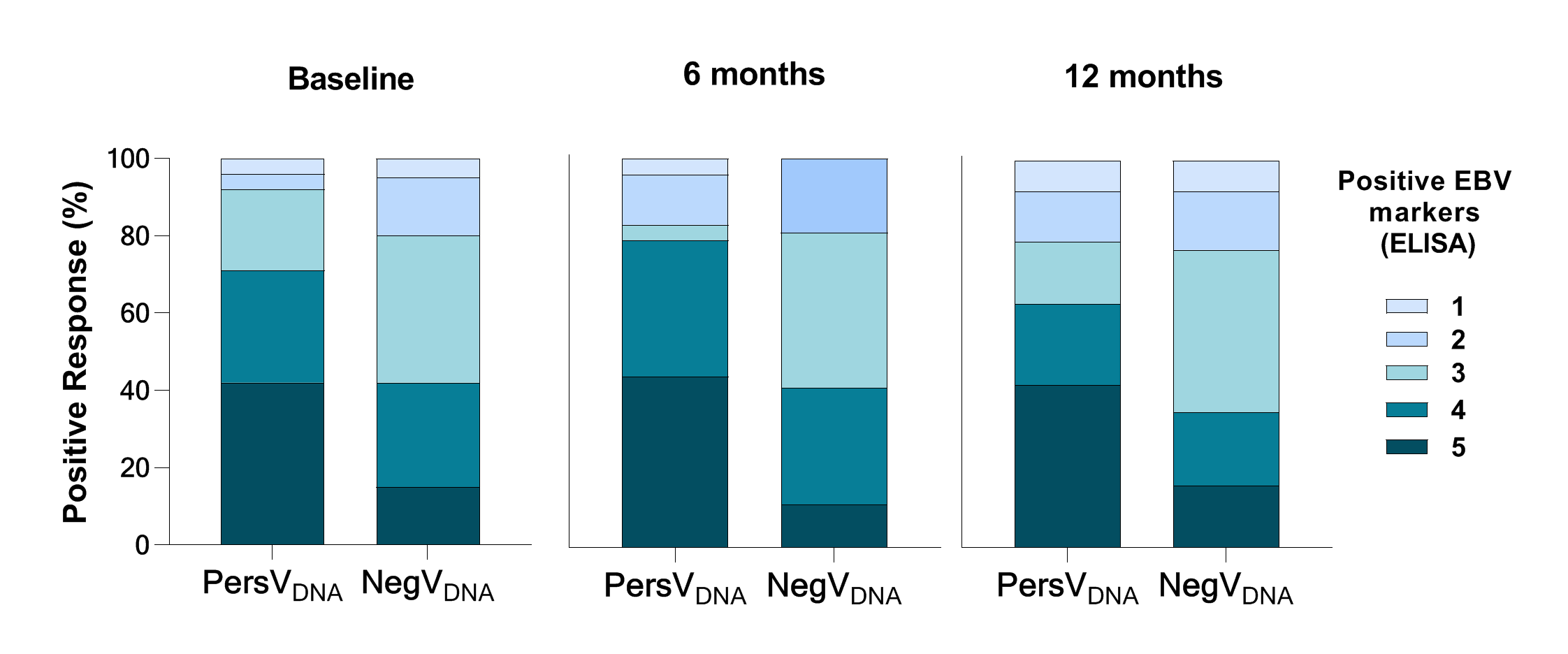

Supplement: S3 Fig — For each group, results were presented as the proportion of responders for one (1), two (2), three (3), four (4) or five (5) EBV serological markers [IgM (VCA-p18, Zebra and EAd-p45/52) and IgG (VCA-p18 and EBNA-1)]. All raw data are available in the S1 Table. (TIF) [file pntd.0010305.s004.tif]

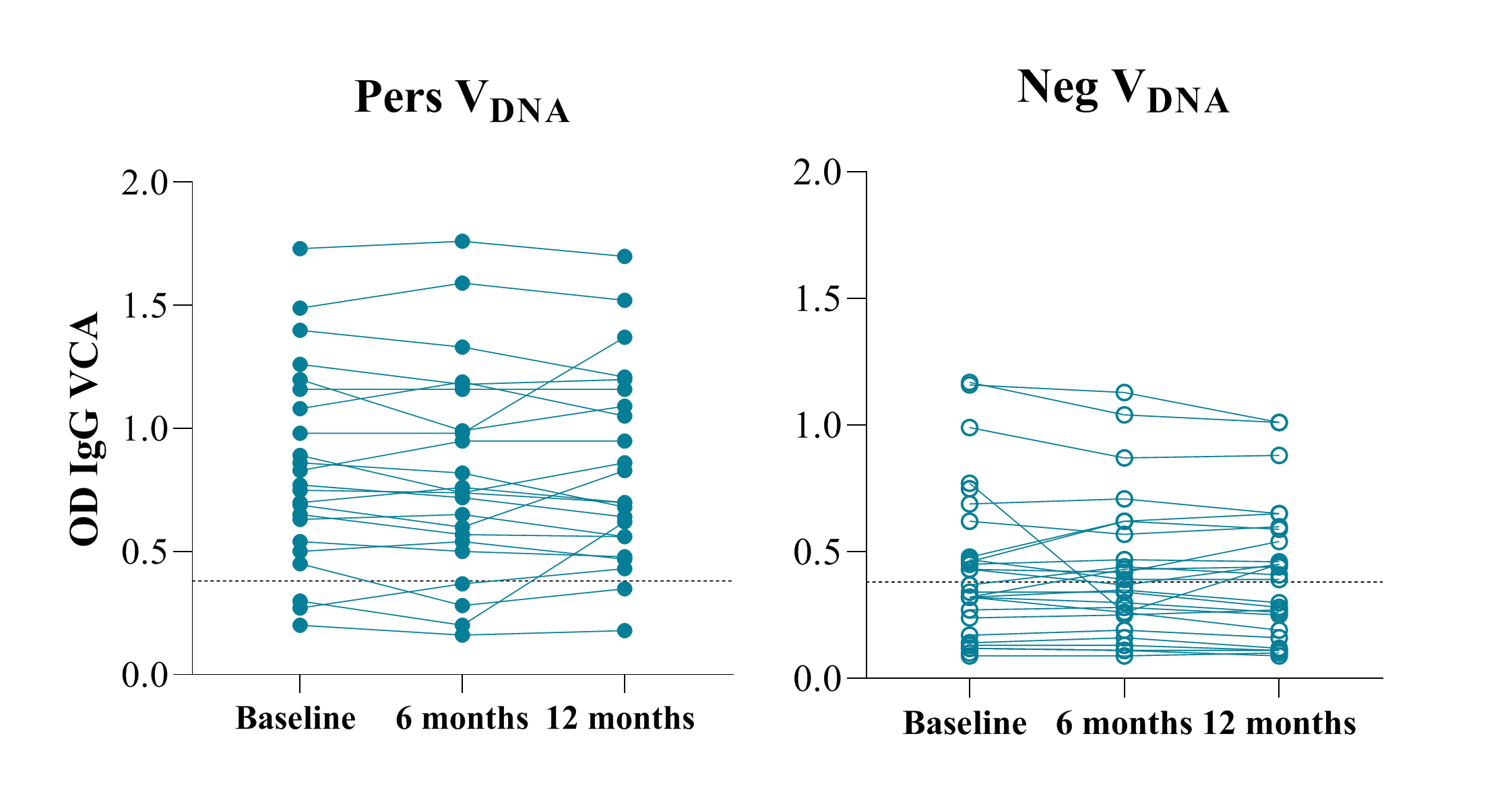

Supplement: S4 Fig — For both graphics, individual data points were expressed as ELISA absorbance (OD) and shown here as a scatter dot plots. Points were connected representing the individual profile over the follow-up period. The dotted line represents the cut-off value that was determined considering an OD > 0.38 as a positive ELISA response (S2 Fig) that classified subjects as low/high responders for VCA-p18. (TIF) [file pntd.0010305.s005.tif]

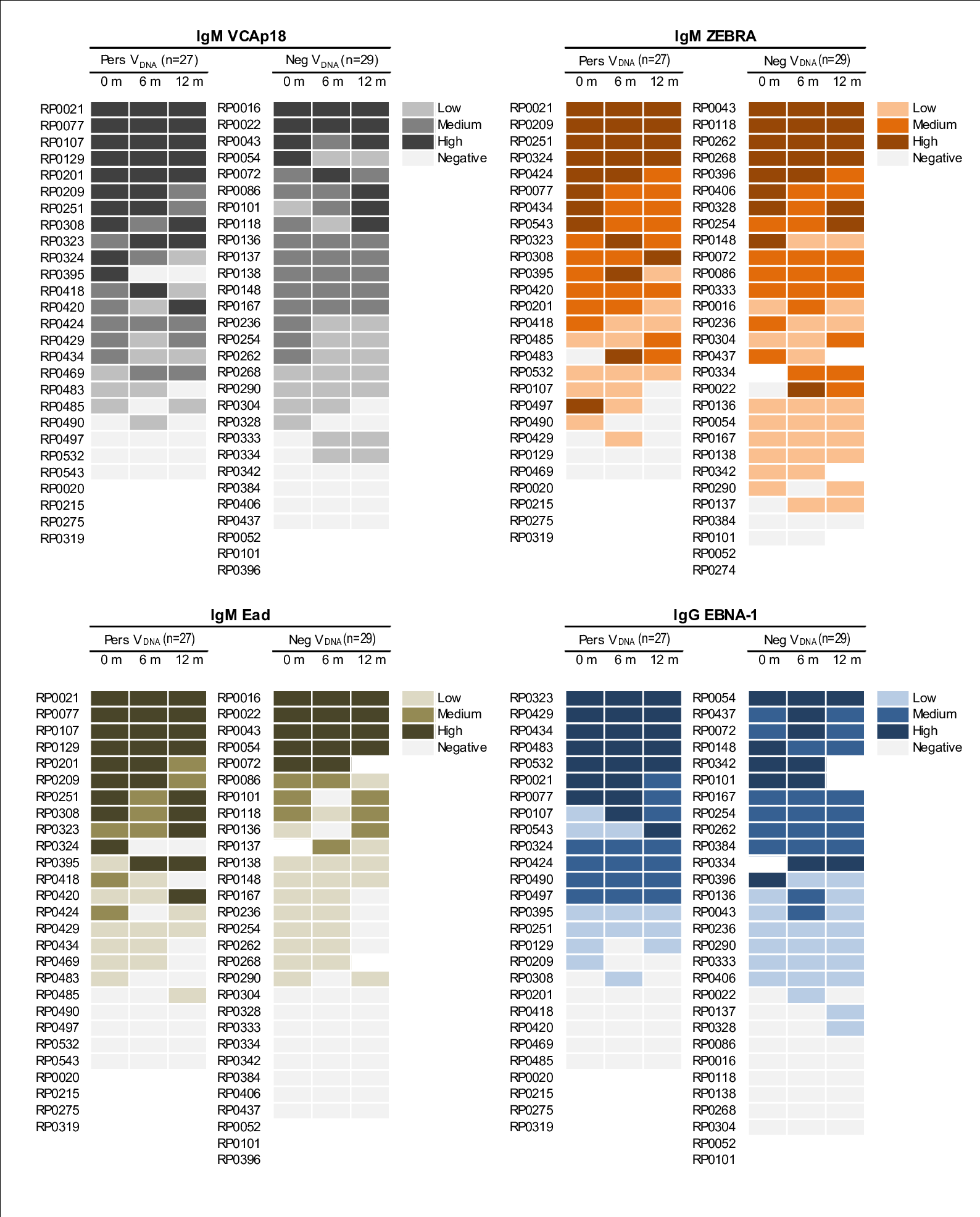

Supplement: S5 Fig — Heatmaps illustrated individual antibody response of each malaria-exposed individuals classified according to the detection (PersVDNA) or not (NegVDNA) of EBV-DNA over the follow-up period. According to EBV antibody response, individuals were categorized as non-responder (negative) or responders (stratified as low, medium or high, according to EBV antibody reactivity). The missing values are recorded as blank spaces. (TIF) [file pntd.0010305.s006.tif]

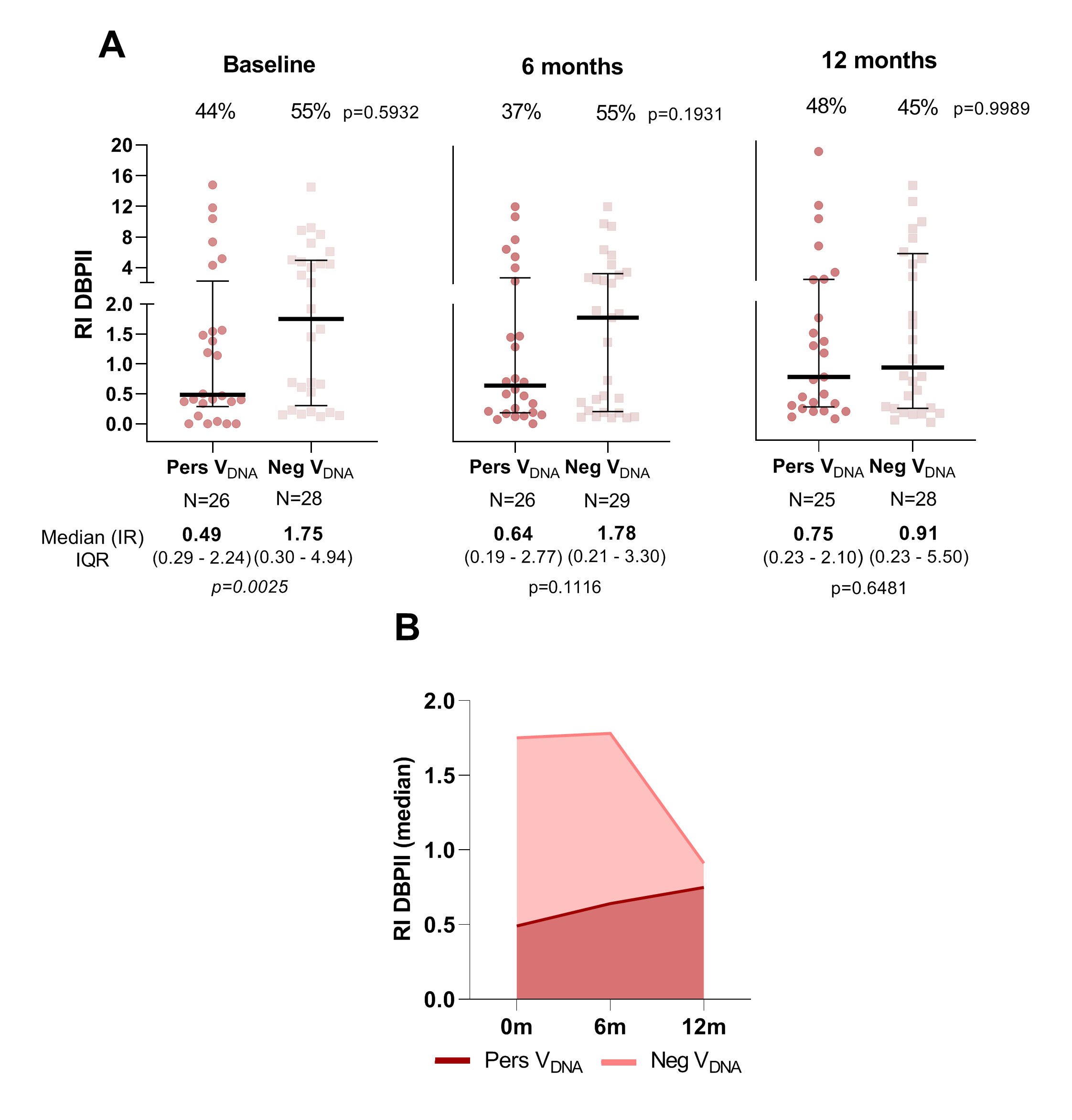

Supplement: S6 Fig — In A, results are shown by cross-sectional surveys (baseline, 6- and 12-month), with individual datapoints expressed as ELISA Reactivity Index (RI) and shown here as a scatter dot plots with lines showing the median with interquartile range (IQR). In B, medians of RIs overtime for each group. Numbers in the top and bottom of each graphic represent the proportion of responders (%) and median (RI) with IQR values, respectively; p-values for significant difference between groups were included and calculated as described in methods. Reactivity Index (RI) was calculated as described in methods and RI > 1 corresponded to an ELISA-positive response. (TIF) [file pntd.0010305.s007.tif]

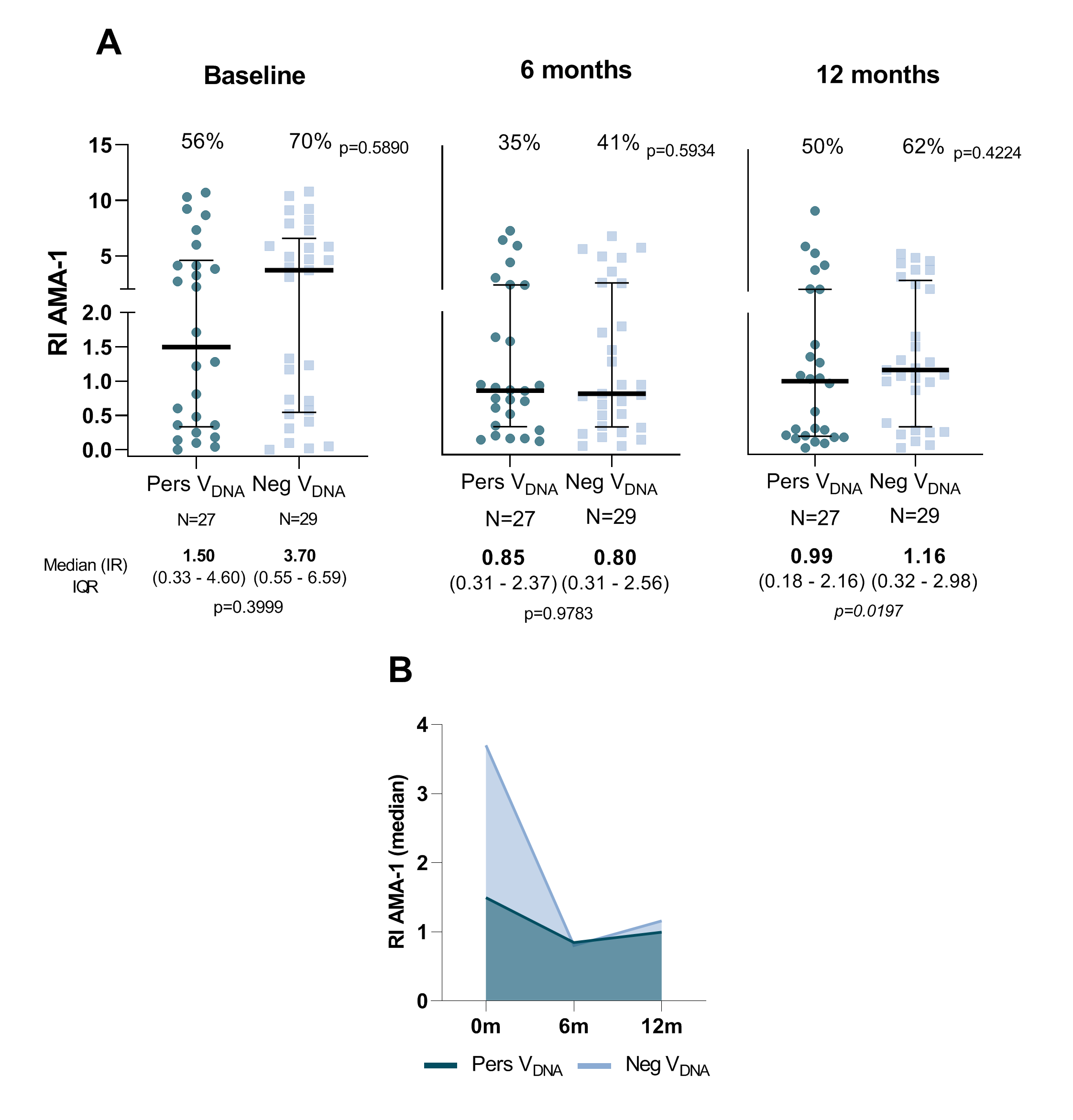

Supplement: S7 Fig — In A, results are shown by cross-sectional surveys (baseline, 6- and 12-month), with individual datapoints expressed as ELISA Reactivity Index (RI) and shown here as a scatter dot plots with lines showing the median with interquartile range (IQR)). In B, medians of RIs overtime for each group. Numbers in the top and bottom of each graphic represent the proportion of responders (%) and median (RI) with IQR values, respectively; p-values for significant difference between groups were included and calculated as described in methods. Reactivity Index (RI) was calculated as described in methods and RI > 1 corresponded to an ELISA-positive response. (TIF) [file pntd.0010305.s008.tif]

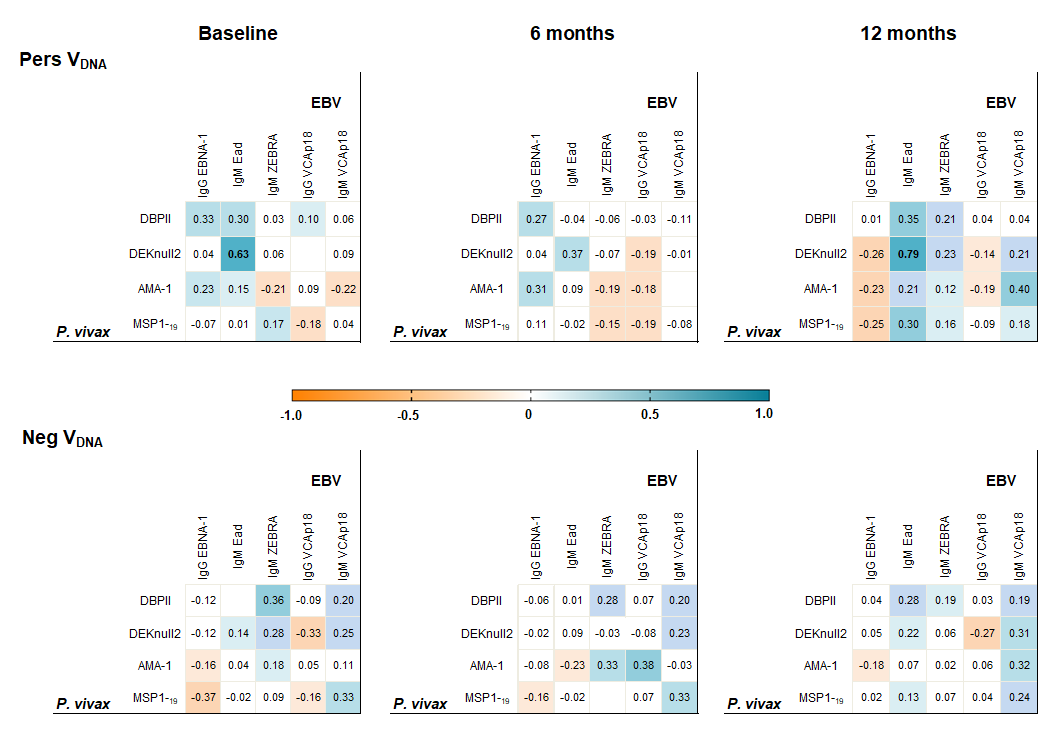

Supplement: S8 Fig — Clustering was based on the Spearman correlation coefficient for assays measuring anti-EBV antibodies in serum. Matrix heatmaps were shown for each cross-sectional survey (baseline, 6- and 12-months), with top and bottom panels representing Pers VDNA an Neg VDNA groups, respectively. Positive correlations shown in blue and negative correlations shown in orange, with numbers in bold statistically significant differences. (TIF) [file pntd.0010305.s009.tif]

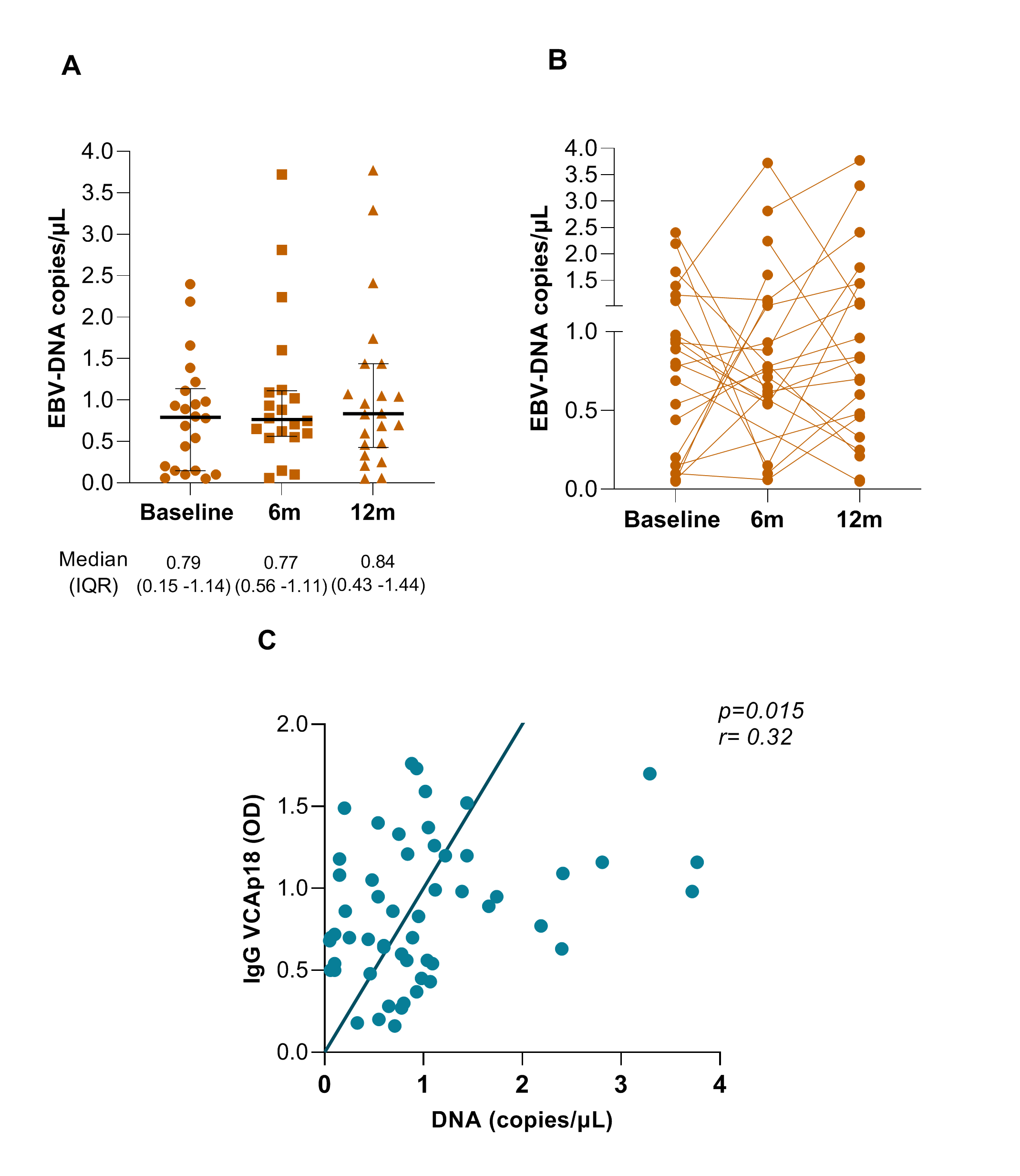

Supplement: S9 Fig — In A, the amplitude of viral DNA is shown by cross-sectional surveys (baseline, 6 and 12 months), with individual data points represented as copy number /μL. Data are shown here as scatterplots with lines showing the median with interquartile range (IQR) (values ​​shown below each cross-section). In B, the dots were connected representing individual variability in EBV-DNA copies overtime, and in C, the association between anti-VCA-p18 IgG antibodies and EBV-DNA copies, as analyzed by the Spearman’s correlation coefficient (r = 0.32 and p<0.05). (TIF) [file pntd.0010305.s010.tif]

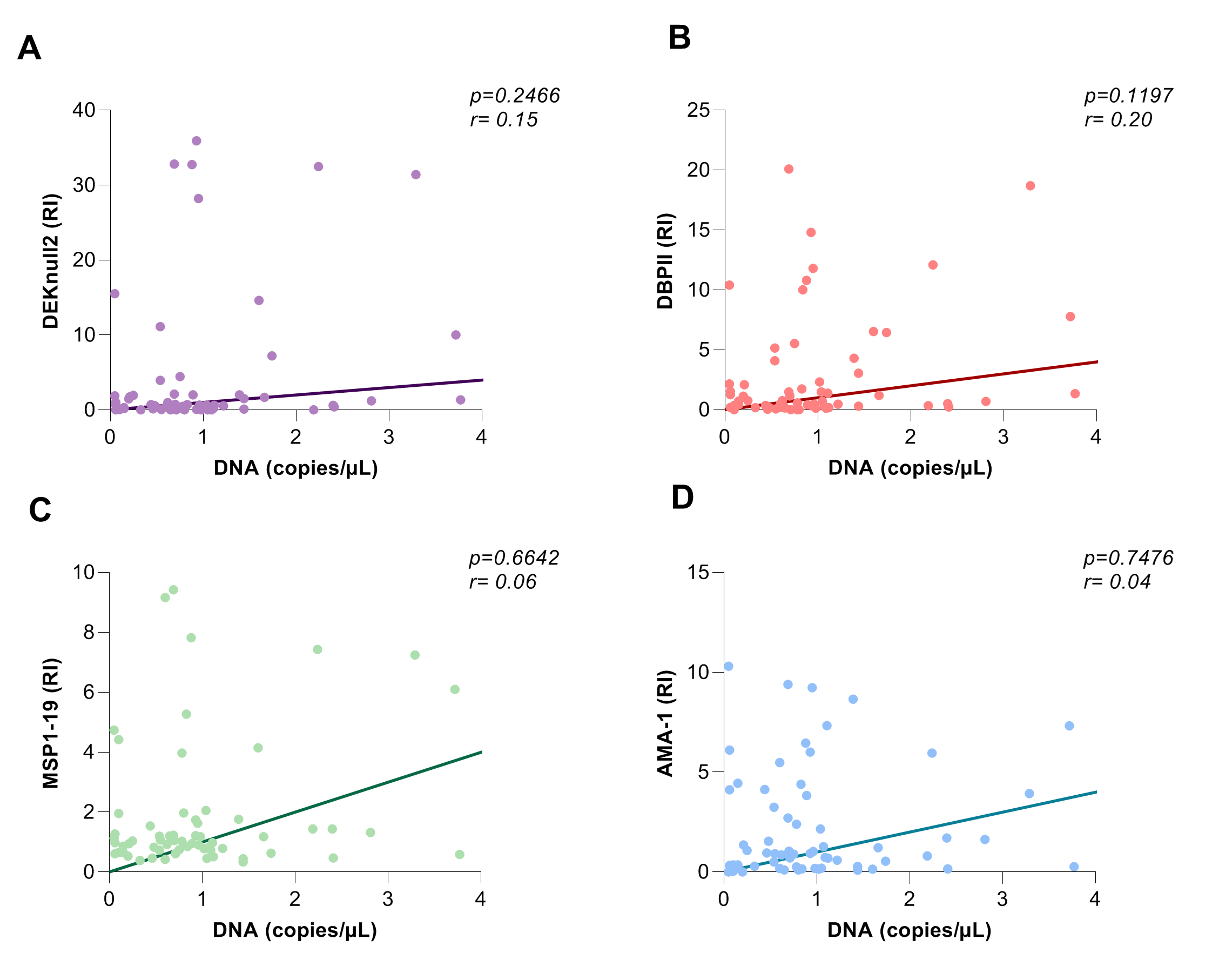

Supplement: S10 Fig — The correlation between antibody levels (reactivity index—RI) to P. vivax blood stage proteins—DEKnull2 (A), DBPII (B), MSP1-19 (C), AMA-1 (D)—and the viral DNA detected (copies/μL) was based on the Spearman correlation coefficient (p>0.05 for all comparison). (TIF) [file pntd.0010305.s011.tif]

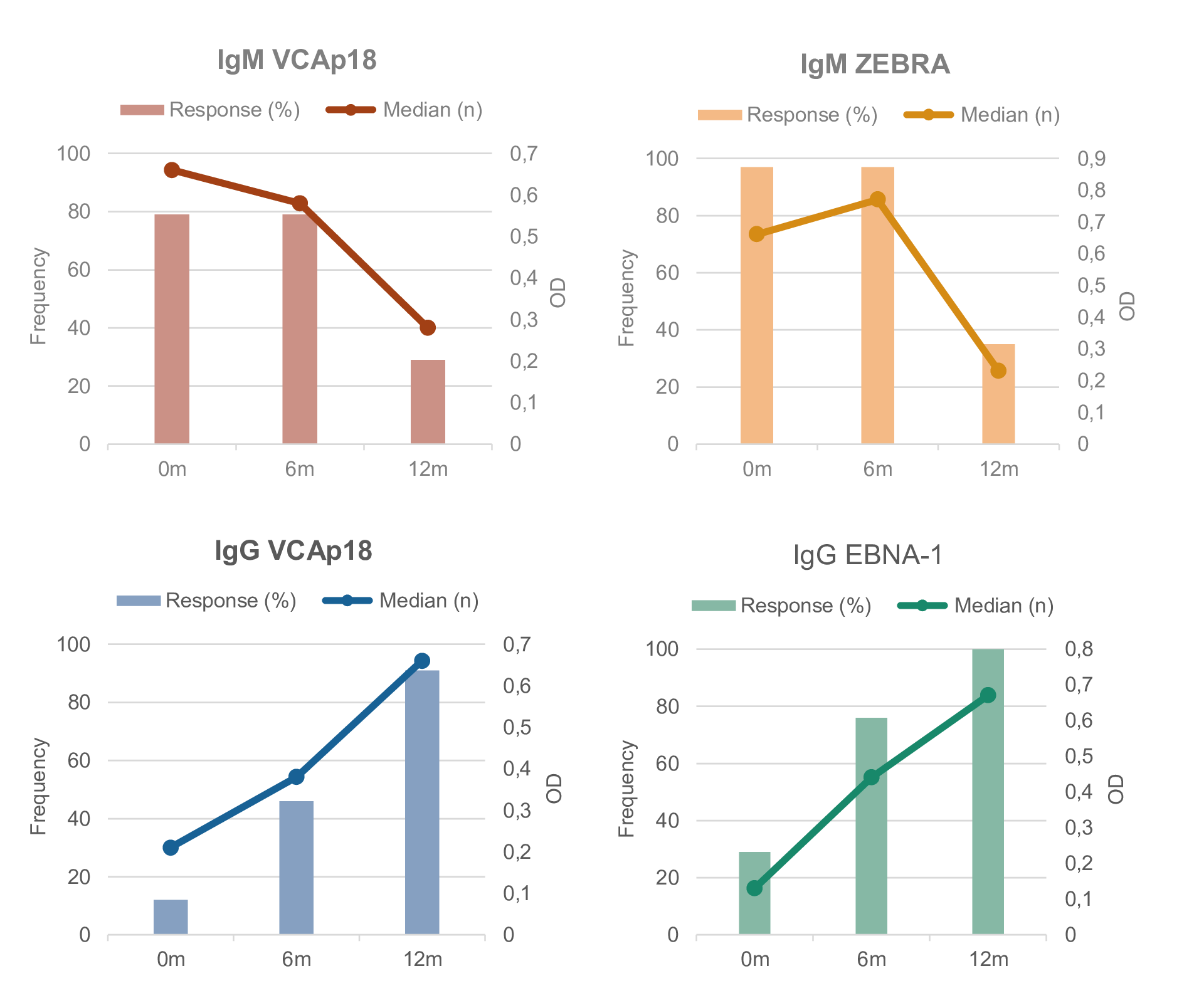

Supplement: S11 Fig — For each EBV-peptide (VCA-p18; Zebra, EAd-p45/52 and EBNA-1) antibody response was represented by frequency of responders (bar) and magnitude of response (optical density-OD median, lines). The results represented three cross-sectional surveys carried-out at 6-month intervals, i.e., at enrollment (0m) and six (6m) and 12-month latter (12m). ELISA assays were carried out as described in the methods. (TIF) [file pntd.0010305.s012.tif]
